# Cataract surgery in patients with Fuchs' endothelial corneal dystrophy

**Published:** 2019-02-10

**Authors:** Soujanya Kaup, Suresh K Pandey

**Affiliations:** 1 Assistant Professor: Yenepoya Medical College, Mangalore, India.; 2 Director: SuVi Eye Institute & Lasik Laser Center, Kota, India.

**Cataract surgery risks corneal decompensation in patients with Fuchs' endothelial corneal dystrophy, but it can also be combined with endothelial keratoplasty to address the condition.**

**Figure:**
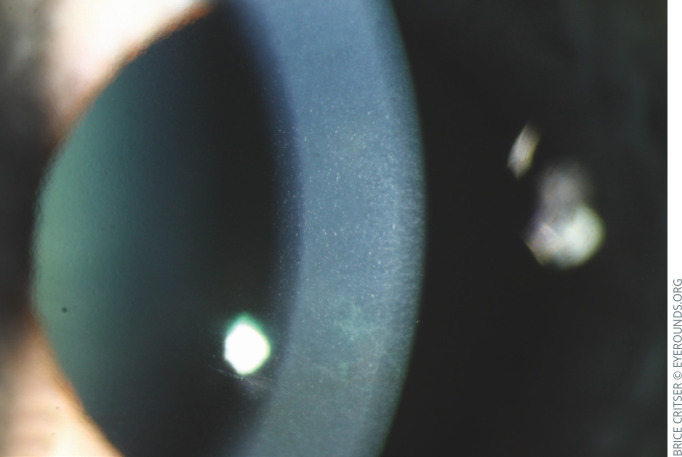
Guttae can have a dimpled or ‘beaten metal’ appearance at the slit lamp.

Fuchs' endothelial corneal dystrophy (FECD) is a progressive disease which mainly affects Descemet's membrane and the endothelium and may eventually lead to corneal decompensation and decreased vision. FECD is treated by performing endothelial keratoplasty: replacement of the diseased inner cell layer of the cornea using donor tissue, e.g., Descemet's stripping endothelial keratoplasty (DSEK).

## Presentation

It is not uncommon to come across patients with both FECD and cataract in clinical practice. The exact prevalence of FECD is not known. It has extreme geographical variability with higher prevalence in European countries and a lower prevalence in South America, Asia and Australia. It is the most prevalent corneal dystrophy in the United States of America.^1^

Patients with FECD often complain of visual loss, which may be attributed to either cataract and/or FECD. Visual loss due to FECD is usually worse in the morning and improves as the day progresses. This is because corneal oedema increases in the night during sleep and reduces as the tear film gradually evaporates during the day. Glare is often a disturbing symptom; it occurs due to confluent guttae or pigment adherent to the endothelium.

## Detection

The presence of guttae is a common sign of endothelial dystrophy. Guttae are excrescences of collagen produced by stressed corneal endothelial cells (see main image above); they form in the middle of the cornea and eventually spread throughout it.

Early cases of FECD may be missed during a cursory slit lamp examination in a busy clinic. Take a careful history of the patient's symptoms and ask whether these are worse in the morning. If possible, examine the eyes under high magnification to detect the condition early.

## Pre-operative considerations

Cataract surgeons are faced with the dilemma of determining whether a cataract operation alone will improve a patient's symptoms, or whether there is a risk that cataract surgery will cause the cornea to decompensate and should therefore be combined with endothelial ketaroplasty. Personalise the treatment plan and consider individual factors such as the cataract density, the health and thickness of the cornea, the anterior chamber depth and the size of the dilated pupil.

The presence of microcystic oedema, stromal thickening and a low central endothelial cell count (less than 1,000 cells/mm^2^), by specular microscopy, indicates an increased likelihood of corneal decompensation after cataract surgery. In these patients, cataract surgery should be combined with endothelial keratoplasty.

In most low- and middle-income countries, a specular microscope might not be widely available. In these situations, central corneal thickness can be used as indirect evidence of endothelial health. This is because dysfunctional endothelial cells are unable to pump water out of the cornea effectively and the corneal stroma swells, thereby increasing the thickness of the central cornea. However, central corneal thickness is variable in the normal population and corneal oedema can still be present in eyes with normal corneal thickness.

In practical terms, central corneal thickness of greater than 640 microns, usually measured using an ultrasound pachymeter, may be predictive of corneal decompensation; this means that a combined operation is needed.^2^

## Patient counselling

Patient counselling is essential for a positive outcome. Allow enough time for counselling and make sure the patient understands that:

- Postoperative recovery time may be longer than usual.
- Endothelial keratoplasty may be required in case of corneal decompensation.
- They will have to come for regular follow-up visits (tell them where to go, and when, and what financial support is available to cover transport costs).

## Technical considerations

### Choice of intraocular lens

If endothelial keratoplasty is combined with cataract surgery, we recommend using a standard monofocal, aspheric intraocular lens (IOL) with larger optic diameter (6.0 mm minimum). Endothelial transplantation introduces negative asphericity and reduced contrast, so avoid the use of multifocal IOLs.

Endothelial transplant patients undergo a hyperopic refractive shift. Thus, where the expectation of decompensation is very high, the IOL power is calculated with a target refraction of -1.25 D. Avoid using an anterior chamber IOL if there is a posterior capsular tear.

### Choice of surgical technique

Both manual small incision cataract surgery (SICS) and phacoemulsification result in a similar amount of endothelial loss.^3^ The choice of surgical technique is hence debatable and depends on the cataract density, the status of cornea and the surgeon's experience and familiarity with each technique. The authors personally prefer phacoemulsification as it has several advantages (small incision, closed anterior chamber and use of phaco power modulation and fluidics to minimise cell loss). Other surgeons, with greater experience of manual SICS than phaco, may prefer the SICS technique. SICS may indeed be the better option when done by surgeons with a great deal of experience in this technique, particularly in patients with very dense cataract, small pupil and compromised zonules.

The newest addition to the cataract surgery technological portfolio is the femtosecond laser, which can be used to create a capsulorrhexis and divide the nucleus into fragments. This option is expensive and has not yet been shown to produce better outcomes than conventional surgery, so it has not become routinely used, even in the most intensively resourced health economies.

### Choice of viscoelastic

A chondroitin sulfate-based dispersive viscoelastic should be used to protect the cornea. It is important to coat the endothelium before doing capsulorhexis. The soft-shell technique, of using a dispersive viscoelastic against the endothelium, followed by a super-cohesive viscoadaptive agent, is particularly effective. Complete removal of viscoelastic must be ensured to prevent a rise in intraocular pressure after surgery.

### Choice of irrigating solution

The choice of solution may also contribute to endothelial cell survival. In units where Ringer's lactate may be the routine choice, Balanced Salt Solution (BSS) would be preferable for FECD patients. BSS, or another solution that contains glutathione, sodium bicarbonate and glucose, will create a more physiologic environment and minimise endothelial cell loss.

**Figure:**
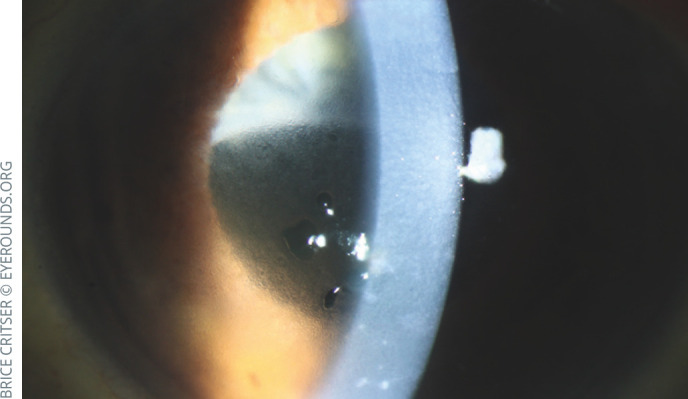
Corneal oedema and bullous keratopathy following cataract surgery. Notice the diffuse epithelial microcysts with several larger bullae visible to the left of the slit beam.

### Wound closure

With compromised endothelial cell function, incisions may not seal securely and placing a suture would be better than excessive corneal stromal hydration.

## Postoperative care

Whatever the technique used to preserve endothelial cells, there is always the risk of prolonged, significant postoperative corneal oedema. These patients may require more frequent steroids and hypertonic saline (e.g. 5% NaCl drops) to help reduce corneal oedema. Periodic follow-up is recommended to evaluate corneal clarity. An excellent visual outcome can be expected in many patients, even those with low pre-operative endothelial counts.^4^

### Take home message

- If central corneal thickness is greater than 640 micrometers, endothelial cells are fewer than 1,000 cells/mm^2^ and/or there is microcystic oedema: consider combining the cataract operation with endothelial keratoplasty.
- Base the choice of cataract surgical technique on the surgeon's experience with the surgical technique, the density of the cataract and the health of the cornea.
- If using phaco, advanced phaco parameters, phaco chop, soft shell technique and the use of a transverse/torsional phaco tip are advocated.
- Pre-operative patient counselling is essential in order to ensure a positive outcome.

**Tips for phaco**

**Minimising endothelial cell loss during phaco**

While performing phacoemulsification, the best way to decrease ultrasonic energy is to use a mechanical method, such as chopping, to break apart the nucleus. Adjust the power modulations to enable the phaco machine to deliver shorter pulses or bursts of ultrasonic energy that minimise endothelial cell loss. Whereas the foot pedal of the phaco machine can deliver blocks of 1 or 2 seconds of energy, the power modulations allows much smaller increments, such as 2 to 4 milliseconds. It is advisable to always use a new phaco tip in these cases and combine lateral phaco (transverse or torsional), with longitudinal phaco.

**When to operate**

All anterior segment intraocular procedures tend to cause at least some loss of endothelial cells. In the early stages, cataracts tend to be less dense and therefore require less ultrasonic energy for phacoemulsification. In the advanced stages of cataract, the ultrasonic energy, combined with the fluid flow through the anterior chamber, may cause a higher degree of endothelial cell loss. It may therefore be appropriate to perform a cataract operation at an earlier stage if phacoemulsification is an option.

**Viscoelastic**

Viscoelastic should be periodically re-injected into the anterior chamber as the irrigation fluid can wash it away during phacoemulsification.

**Irrigation**

The flow of irrigating solution can create currents against the endothelial cells, inducing damage. Use low-flow parameters, make snug incisions and stay away from the corneal endothelium.

*With thanks to EyeRounds.org for their generous contribution of images for this article.* ***www.eyerounds.org***
